# Unraveling the Role of Hypothyroidism in Non-alcoholic Fatty Liver Disease Pathogenesis: Correlations, Conflicts, and the Current Stand

**DOI:** 10.7759/cureus.14858

**Published:** 2021-05-05

**Authors:** Rajvi Gor, Nabeel A Siddiqui, Ransirini Wijeratne Fernando, Archana Sreekantan Nair, Janan Illango, Mushrin Malik, Pousette Hamid

**Affiliations:** 1 Research, California Institute of Behavioral Neurosciences & Psychology, Fairfield, USA; 2 Neurology, California Institute of Behavioral Neurosciences & Psychology, Fairfield, USA

**Keywords:** non-alcoholic fatty liver disease (nafld), hypothyroidism, nafld pathophysiology, thyroid dysfunction, thyroid-stimulating hormone (tsh), thyroxine (t4), thyroid hormones, non-alcoholic steatohepatitis (nash), hypothyroidism-induced nafld, nafld causes

## Abstract

Non-alcoholic fatty liver disease (NAFLD) has become one of the most common causes of chronic liver diseases globally. Because thyroid hormones play a crucial role in lipid metabolism, thyroid dysfunction has been implicated in NAFLD pathogenesis in the past decade, with hypothyroidism-induced NAFLD being regarded as a distinct disease entity. However, there has been no common consensus yet, and several studies have found contradictory results. Hence, we conducted this systematic review to represent the current view on the role of hypothyroidism (HT) and individual thyroid function parameters such as thyroid-stimulating hormone (TSH), thyroxine (T4), triiodothyronine (T3), thyroid peroxidase antibody (TPOAb), and thyroglobulin antibody (TGAb) in NAFLD pathogenesis. We searched PubMed, PubMed Central, and Semantic Scholar databases from inception until January 2021 to identify relevant observational (case-control, cross-sectional, and longitudinal) studies. A total of 699 articles were recognized through our database search. After applying the eligibility criteria and performing quality assessment, 10 studies involving 42,227 participants were included in the final systematic review. Each of these studies assessed different thyroid function parameters, and NAFLD was found to be associated with HT in two studies, elevated TSH in three studies, suppressed T4 in three studies, elevated T3 in one study, and elevated TPOAb in one study. There was also a wide heterogeneity in HT definition, study population characteristics, and study design among these studies, making a direct comparison difficult. Because the recognition of HT-induced NAFLD has possible diagnostic, therapeutic, and prognostic implications, we recommend that comprehensive, long-term prospective studies be carried out to determine if HT or thyroid function parameters are causally associated with NAFLD.

## Introduction and background

Steeply rising from a worldwide prevalence of 8.2% in 1990 [1] to 25% in 2019 [2], non-alcoholic fatty liver disease (NAFLD) has surfaced as one of the most prominent causes of chronic liver diseases. Apart from its hepatic complications of cirrhosis and hepatocellular carcinoma (HCC), the disease burden of NAFLD also stems from its broad spectrum of extrahepatic comorbidities such as cardiovascular disease (CVD), type 2 diabetes mellitus (T2DM), obesity, chronic kidney disease (CKD), and obstructive sleep apnea [3]. NAFLD is defined by the presence of >5% steatotic hepatocytes in a liver biopsy specimen in the absence of alcohol consumption, viral, drug-induced, autoimmune, and genetic etiologies [4,5]. The umbrella term NAFLD includes the entire range of liver involvement from simple steatosis to non-alcoholic steatohepatitis (NASH), advanced fibrosis, and cirrhosis, which can progress to HCC. Moreover, NAFLD has been predicted to become the most important indication for liver transplantation in the future [6]. The widely known risk factors for NAFLD include metabolic syndrome determinants such as obesity, hyperlipidemia, and insulin resistance [7]. However, recently, NAFLD has also been linked with endocrine disorders such as hypothyroidism (HT), polycystic ovary syndrome, hypogonadism, and growth hormone deficiency [8].

While the recognition of NAFLD as a growing epidemic has been widely acknowledged, the association between NAFLD and HT has only recently become a topic of attention. HT, depending on its etiology, can be primary (due to thyroid gland dysfunction), secondary (due to pituitary dysfunction), or tertiary (due to hypothalamic dysfunction). Based on thyroid-stimulating hormone (TSH) and serum free thyroxine (FT4) levels, primary HT can be further categorized as subclinical (elevated TSH, normal range serum FT4) or overt (elevated TSH, low serum FT4). Because thyroid hormones are one of the principal drivers of fat and cholesterol metabolism in the human biome [9], their deficiency could be pathophysiologically associated with NAFLD, which on various occasions has been considered to be a precedent, comorbidity, or the result of metabolic syndrome [8]. Although a common consensus is yet to be reached, the term HT-induced NAFLD has recently been recognized as a discrete disease entity [10].

The identification and better understanding of this association between HT and NAFLD could open up a novel modality for diagnosis, prognosis, and management of NAFLD. In a study involving NAFLD patients with chronic hepatitis B, elevated serum TSH level was found to be an independent predictive factor of incident NASH [11], raising the question if TSH could be a possible marker of disease severity. Currently, NAFLD treatment focuses on weight loss for overweight or obese patients, alcohol abstinence, immunizations, and cardiovascular risk factor (hypertension [HTN], hypercholesterolemia, and diabetes mellitus [DM]) modification. By virtue of their role in metabolism, thyroid hormones have been shown to have anti-steatotic effects in in-vitro and in-vivo mammalian models [12]. In addition, in patients with concomitant NASH and Graves’ disease, an increase in thyroid hormone levels was associated with a decrease in liver enzyme levels [13]. If NAFLD-associated HT is found to be reversible by correction of underlying thyroid hormone deficiency, it could imply a more favorable prognosis for this subset of NAFLD patients.

As the concept of HT-induced NAFLD is newly emerging and management guidelines are yet to be defined, HT is often not evaluated in patients with NAFLD and vice versa. This makes it even more necessary to increase awareness regarding this emerging association among practicing physicians, gastroenterologists, and endocrinologists. In this systematic review, we aim to review the studies analyzing the relationship between HT and NAFLD conducted in the adult human population by collecting information from previously published articles and discussing the proposed pathophysiologic mechanisms to understand this relationship better.

## Review

Methods

This systematic review was conducted in accordance with the Preferred Reporting Items for Systematic Reviews and Meta-Analyses (PRISMA) guidelines [14].

Databases and Search Strategy

We conducted a systematic search of articles published in PubMed, PubMed Central (PMC), and Semantic Scholar online databases from inception until January 19, 2021. The search strategy for our research question on PubMed, using Medical Subject Heading (MeSH) terms and keywords, was as follows: (“Non-alcoholic fatty liver disease” OR “NAFLD” OR “NASH” OR “Non-alcoholic steatohepatitis” OR “Non-alcoholic Fatty Liver Disease” [Mesh]) AND (“hypothyroidism” OR “thyroid” OR “thyroid hormone” OR “thyromimetics” OR “thyroid dysfunction” OR “thyroid-stimulating hormone” OR “thyroxine” OR “triiodothyronine” OR “Thyroid Hormones” [Mesh] OR “Hypothyroidism” [Mesh]).

Eligibility Criteria and Study Selection

Two researchers (RG and NS) independently screened each article’s title and abstract to determine eligibility. The following inclusion criteria were utilized to screen the results: (1) observational (cross-sectional, case-control, longitudinal) studies that explored the relationship between HT or thyroid function parameters and NAFLD; (2) studies published from inception until January 19, 2021; (3) free full-text articles available; (4) studies published in the English language; and (5) studies involving adult human population irrespective of gender, ethnicity, or study location. The exclusion criteria were as follows: (1) editorials, manuscripts, case reports, posters, literature reviews, systematic reviews, meta-analyses, animal studies, and clinical trials; (2) studies including patients with excessive alcohol intake, hepatitis-B (HBV) or hepatitis-C (HCV) virus infection, drug or toxin-induced liver injury, or any other concomitant liver disease other than NAFLD; (3) studies involving pediatric subjects; (4) irrelevant studies; (5) duplicate studies; and (6) studies where the diagnosis of NAFLD was solely based on biochemical tests and indices (such as hepatic steatosis index, fatty liver index, and others) without any imaging or biopsy. The full-text articles of studies that met the above inclusion and exclusion criteria were evaluated for eligibility in the final review.

Data Extraction

Two reviewers (RG and NS) independently extracted data from each study utilizing a standardized data extraction tool. The following parameters were recorded: study design, study location, study duration, sample size, study population characteristics, methods for assessing NAFLD and thyroid status, thyroid function parameters assessed, NAFLD prevalence and outcomes assessed, covariates adjusted, and conclusion.

Risks of Bias and Quality Assessment

Two researchers (RG and NS) independently performed the quality appraisal of eligible studies. The following quality assessment tools were utilized to appraise individual studies critically: Appraisal tool for cross-sectional studies (AXIS) scale for cross-sectional studies and Newcastle-Ottawa scale (NOS) for case-control and cohort studies. The AXIS scale includes 20 items divided into five categories, namely, Introduction, Methods, Results, Discussion, and Others. Eight items, divided into three categories, namely, Selection, Comparability, Exposure, or Outcome, are included in the NOS scale. Only high and fair quality studies suitable to the aims of our research question and that met the specified eligibility criteria were included in the final review.

Results

Literature Search and Study Selection

**Figure 1:**
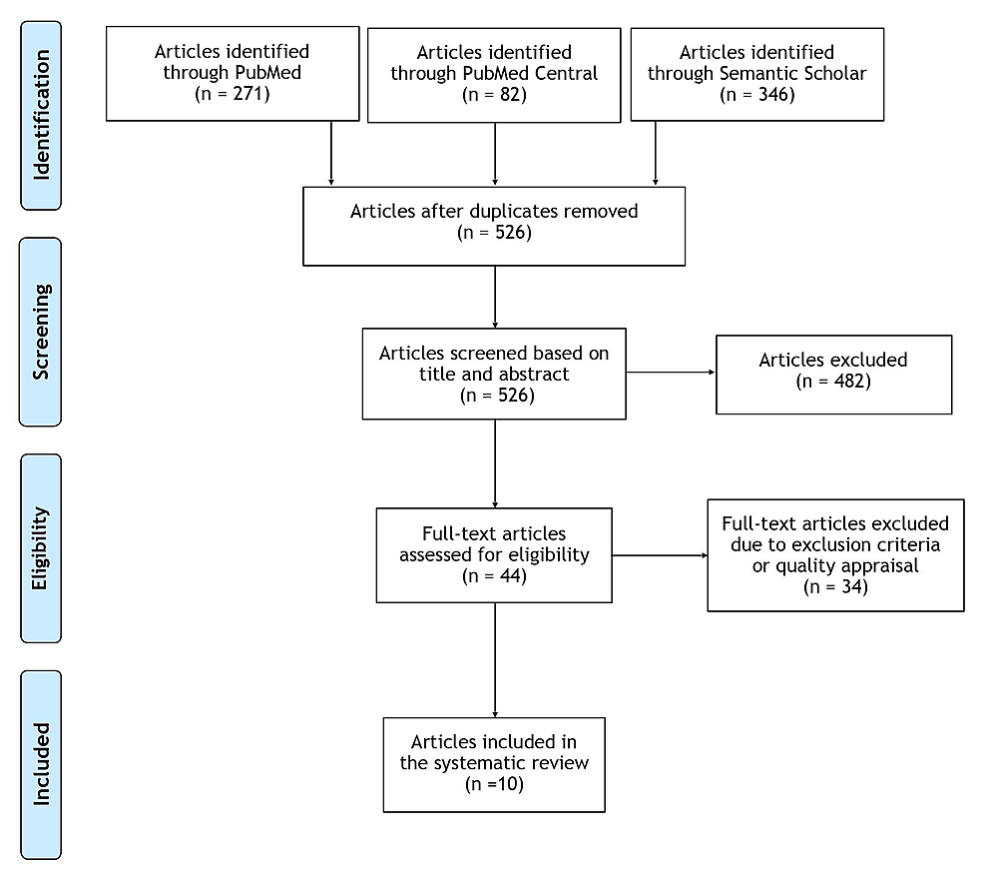
The PRISMA flow diagram. PRISMA: Preferred Reporting Items for Systematic Reviews and Meta-Analyses

Figure 1 summarizes our literature search results and the study selection process. Our search strategy yielded a total of 699 articles: 271 from PubMed, 82 from PMC, and 346 from Semantic Scholar. After manually removing 173 duplicates, 526 articles were identified and independently screened by two researchers (RG and NS) based on title and abstract. After applying inclusion and exclusion criteria, 44 full-text articles were found to be potentially eligible. Of these, 10 articles [15-24] were identified as suitable to the aims of our research question and met the quality specifications necessary for this systematic review.

Study Characteristics

Table 1 summarizes the main characteristics of the 10 included studies. Of these, seven were cross-sectional studies, two were retrospective cohort studies, and one was a prospective cohort study. All of these studies were published between 2011 and 2021. The following studies were included: Janovsky et al. [15], Tahara et al. [16], Wang et al. [17], Bano et al. [18], Eshraghian et al. [19], Gökmen et al. [20], Lee et al. [21], Ludwig et al. [22], Mazo et al. [23], and Shi et al. [24]. Five of these studies were conducted in Asia, two in South America, two in Europe, and one in the Middle East. A total of 42,227 individuals were assessed across all 10 studies, of which 23,484 (55.61%) were males and 18,743 (44.39%) were females. The study participants were selected from the general population in three studies, from patients coming for routine health check-ups in two studies, and from patients admitted to outpatient clinics or hospitals in five studies. NAFLD was detected by ultrasound in nine studies and liver biopsy in one study. HT was diagnosed based on serum TSH levels with or without thyroxine (T4) and triiodothyronine (T3) levels in seven studies, based on the history of T4 replacement therapy in patients diagnosed with HT for more than one year in one study, and remained undefined in two studies. Six studies evaluated the association between NAFLD and HT itself. Some studies also analyzed the relationship between NAFLD risk and individual thyroid function parameters such as TSH was assessed in nine studies, T4 in eight studies, T3 in four studies, thyroid peroxidase antibody (TPOAb) in four studies, and thyroglobulin antibody (TGAb) in two studies.

**Table 1:** Main characteristics of included studies. NAFLD: non-alcoholic fatty liver disease; TSH: thyroid-stimulating hormone; T4: thyroxine; FT4: free thyroxine; TT4: total thyroxine; T3: triiodothyronine; TT3; total triiodothyronine; TPOAb: thyroid peroxidase antibody; TGAb: thyroglobulin antibody; SCH: subclinical hypothyroidism; OHT: overt hypothyroidism; T2DM: type 2 diabetes mellitus

Study	Study design	Sample size	Study population characteristics	NAFLD diagnosis	Hypothyroidism diagnosis	Thyroid function parameters assessed			
						TSH	T4	T3	TPOAb/TGAb
Janovsky et al. [15] (2018, Brazil)	Cross-sectional	10, 539	Euthyroid subjects presenting for routine health check-up (72.56% males)	Ultrasound	Only euthyroid subjects chosen	Yes	-	-	-/-
Tahara et al. [16] (2019, Japan)	Cross-sectional	140	70 patients with SCH and 70 euthyroid age- and sex-matched controls selected from patients not taking thyroid hormones or anti-thyroid drugs and undergoing thyroid hormone assessment (51.42% males)	Ultrasound	SCH: serum TSH >4.00 μU/L and FT4 ranging 0.90-1.80 ng/dL	Yes	Yes	-	-/-
Wang et al. [17] (2020, China)	Cross-sectional	400	Hospitalized patients with T2DM (46.25% males)	Ultrasound	SCH: serum TSH >4.94 uIU/mL and FT4 within the reference range. OHT: serum TSH >4.94 uIU/mL and FT4 <0.7 ng/dL	Yes	Yes	-	Yes/Yes
Bano et al. [18] (2016, Netherlands)	Prospective cohort	9,419	Subjects selected from Rotterdam Study, a population-based cohort study (43.51% males)	Ultrasound (FLI at baseline)	SCH: serum TSH >4.0 mIU/L and FT4 ranging 0.85-1.95 ng/dL. OHT: serum TSH >4.0 mIU/L and FT4 levels <0.85 ng/dL	Yes	Yes	-	Yes/-
Eshraghian et al. [19] (2013, Iran)	Cross-sectional	832	Healthy adult subjects selected by clustered random sampling from a town (38.7% males)	Ultrasound	SCH: serum TSH >5.2 mIU/L and FT4 ranging 11.5-23 pmol/L. OHT: TSH >5.2 mIU/L and FT4 levels <11.5 pmol/L	Yes	Yes	Yes	Yes/Yes
Gokmen et al. [20] (2016, Turkey)	Cross-sectional	115	Patients admitted to the outpatient clinic for routine care (34.78% males)	Ultrasound	Serum TSH ≥4.1 mIU/L	Yes	Yes	Yes	-/-
Lee et al. [21] (2015, Korea)	Retrospective cohort	18,544	Subjects presenting for medical health check-up (53.26% males)	Ultrasound	SCH: serum TSH >4.2 m IU/L and FT4 ranging 0.97-1.68 ng/dL. OHT: serum TSH >4.2 mIU/L and FT4 <0.97 ng/dL	Yes	Yes	-	-/-
Ludwig et al. [22] (2015, Germany)	Cross-sectional	1276	Subjects not taking iodine or thyroid hormones or anti-thyroid medicines were randomly selected from a city by Municipal Registry staff (52.82% males)	Ultrasound	SCH: TSH ≥34 IU/mL; TT4: 12.8-20.4 pmol/L; and TT3: 3.92-6.74 pmol/L OHT: TSH ≥34 IU/mL and TT4 <12.8 pmol/L	Yes	Yes	Yes	Yes/-
Mazo et al. [23] (2011, Brazil)	Retrospective cohort	103	NAFLD patients who were followed at Hepatology Outpatient Unit were divided into NASH and steatosis-only groups (30% males)	Liver biopsy	Patients diagnosed with hypothyroidism for >1 year and receiving synthetic T4 replacement therapy	-	-	-	-/-
Shi et al. [24] (2021, China)	Cross-sectional	859	Hospitalized patients with T2DM (62.40% males)	Ultrasound	Not defined	Yes	Yes	Yes	-/-

Hypothyroidism and Risk of Non-alcoholic Fatty Liver Disease

Out of 10 studies, six [16,18-21,23] evaluated the association between HT and NAFLD risk. Tahara et al. [16] observed that subclinical hypothyroidism (SCH) was an independent risk factor (odds ratio [OR] = 4.74; 95% confidence interval [CI]: 10.91-12.91; p = 0.001) of NAFLD after multivariate adjustment for metabolic syndrome risk factors (body mass index [BMI], high-density lipoprotein-cholesterol [HDL-C], triglyceride, HTN, DM). In addition, the proportion of patients with a Fibrosis-4 (FIB-4) index value of ≥2.6, suggesting a higher risk of liver fibrosis, was significantly higher (p = 0.008) in subclinical hypothyroid patients (34.3%) than in euthyroid patients (12.9%). Bano et al. [18] found that HT (subclinical and overt) was independently associated with increased NAFLD risk (OR = 1.24; 95% CI: 1.01-1.53; p < 0.05) after covariate adjustment for age, sex, alcohol intake, smoking, BMI, HTN, DM, total cholesterol, triglycerides, use of hypolipidemic drugs, cohort, and follow-up time. Also, SCH (OR = 2.14; 95% CI: 1.04-4.07; p < 0.05) and overt hypothyroidism (OHT) (OR = 6.64; 95% CI: 1.04-23.98; p < 0.05) were significantly associated with an increased risk of liver fibrosis (liver stiffness ≥ 8.0 kPa on transient elastography) among NAFLD patient group. On the other hand, Eshraghian et al. [19] noted that neither SCH (OR = 1.12; 95% CI: 0.51-2.46; p > 0.05) nor OHT (OR = 0.87; 95% CI: 0.33-2.28; p > 0.05) were significantly associated with NAFLD. Gokmen et al. [20] observed no statistically significant difference (p = 0.819) in NAFLD prevalence between euthyroid and hypothyroid groups. Lee et al. [21] found that there was no significant difference (p = 0.132) between the overall incidence of NAFLD in euthyroidism (ET) (12.8%), SCH (11%), and OHT (12.7%) patients. Even after multivariate adjustment for metabolic syndrome risk factors, neither SCH (adjusted hazard ratio [aHR] = 0.965; 95% CI: 0.814-1.143; p = 0.678) nor OHT (aHR = 1.255; 95% CI: 0.83-1.89; p = 0.282) was an independent predictor of incident NAFLD. Mazo et al. [23] discovered that although HT was positively correlated with insulin levels (correlation index [r] = 0.213; p = 0.03), homeostasis model assessment for insulin resistance (HOMA-IR) index (r = 0.221; p = 0.02), aspartate aminotransferase (AST) (r = 0.234; p = 0.01), and triglyceride (r = 0.233; p = 0.01) levels in patients with NAFLD; there was no significant correlation (p > 0.05) between HT and occurrence of NASH among patients with biopsy-proven NAFLD.

Thyroid-Stimulating Hormone Levels and Risk of Non-alcoholic Fatty Liver Disease

Out of 10 studies, nine [15-22,24] evaluated the association between TSH levels and NAFLD risk. Janovsky et al. [15] detected that among euthyroid individuals, higher TSH values were associated with higher NAFLD prevalence (OR = 1.22; p < 0.01) after adjusting for age and gender. However, this association became insignificant (OR = 0.93; p = 0.20) when additionally adjusted for metabolic syndrome characteristics (abdominal circumference, triglycerides, HDL-C, blood pressure, fasting glucose) and smoking. Tahara et al. [16] observed that TSH elevation (OR = 1.12; 95% CI: 1.01-1.40; p = 0.033) was an independent risk factor of NAFLD after multivariate adjustment for metabolic syndrome risk factors (BMI, HDL-C, triglyceride, HTN, DM). Wang et al. [17] found that hospitalized T2DM patients with NAFLD had significantly higher TSH levels (p = 0.02) than T2DM patients without NAFLD. Bano et al. [18] noted that increased TSH levels were significantly (OR = 1.09; 95% CI: 1.01-1.19; p < 0.05) associated with NAFLD risk after adjusting for age, sex, cohort, alcohol intake, smoking, and follow-up time; however, this association became statistically insignificant (OR = 1.07; 95% CI: 0.98-1.17; p ≥ 0.05) after further adjustment for total cholesterol, triglycerides, BMI, HTN, DM, and use of hypolipidemic drugs. In addition, among NAFLD patients, increased TSH levels (OR = 1.49; 95% CI: 1.04-2.15) were significantly associated with an increased risk of liver fibrosis (liver stiffness ≥ 8.0 kPa on transient elastography). Eshraghian et al. [19] discovered that there was no significant difference (p > 0.05) in TSH levels between subjects with NAFLD compared to those without NAFLD. Gokmen et al. [20] found no significant difference in TSH (p = 0.138) levels between NAFLD and non-NAFLD groups. Similarly, Lee et al. [21] observed no statistically significant difference in TSH (p = 0.8) levels between NAFLD and non-NAFLD groups. Ludwig et al. [22] noted that TSH levels (OR = 0.992; CI: 0.945-1.042; p = 0.7453) were not significantly associated with NAFLD risk. Shi et al. [24] found that there was no significant difference in TSH levels (p > 0.05) between NAFLD and non-NAFLD groups.

Thyroxine Levels and Risk of Non-alcoholic Fatty Liver Disease

A total of eight studies [16-22,24] evaluated the role of T4 levels in NAFLD risk. Tahara et al. [16] observed that FT4 levels were not significantly associated with NAFLD (OR = 0.12; 95% CI: 0.01-1.31; p = 0.083) risk. Wang et al. [17] noted that T2DM patients with NAFLD had significantly lower FT4 levels (p < 0.001) compared to T2DM patients without NAFLD. Bano et al. [18] found that decreased FT4 levels (OR = 0.42; 95% CI: 0.28-0.63; p < 0.05) were significantly associated with increased NAFLD risk even after covariate adjustment for age, sex, alcohol intake, smoking, BMI, HTN, T2DM, total cholesterol, triglycerides, use of hypolipidemic drugs, cohort, and follow-up time. Eshraghian et al. [19] noted that there was no significant difference (p > 0.05) in FT4 levels between subjects with NAFLD compared to those without NAFLD. Gokmen et al. [20] detected no significant difference in FT4 (p = 0.025) levels between NAFLD and non-NAFLD groups. Similarly, Lee et al. [21] observed no statistically significant difference in FT4 (p = 0.988) levels between NAFLD and non-NAFLD groups. On the contrary, Ludwig et al. [22] found that total T4 (TT4) levels were significantly associated with NAFLD risk (OR = 0.987; 95% CI: 0.979-0.995; p = 0.0008). This association between TT4 and NAFLD remained significant after adjusting for age (OR = 0.990; 95% CI: 0.982-0.998; p = 0.0143) or BMI (OR = 0.988; 95% CI: 0.979-0.997; p < 0.001); but not after adjusting for BMI, age, waist to hip ratio, and gender together (OR = 0.994; 95% CI: 0.984-1.005; p = 0.2959). Shi et al. [24] noted no significant difference (p > 0.05) in FT4 levels between NAFLD and non-NAFLD groups.

Triiodothyronine Levels and Risk of Non-alcoholic Fatty Liver Disease

Out of 10 studies, four [19,20,22,24] evaluated the association between T3 levels and NAFLD risk. Eshraghian et al. [19] noted no significant difference (p > 0.05) in free T3 (FT3) levels between subjects with NAFLD compared to those without NAFLD. Likewise, Gokmen et al. [20] observed no significant difference in FT3 (p = 0.479) levels between NAFLD and non-NAFLD groups. However, elevated FT3/FT4 ratio was an independent risk factor for NAFLD in combined ET and SCH patients (OR = 1.834; 95% CI: 1.089-3.569; p = 0.02) as well as SCH patients alone (OR = 3.540; 95% CI: 1.309-9.575; p = 0.01) after adjusting for waist circumference, triglycerides, total cholesterol, uric acid, and HOMA-IR. Ludwig et al. [22] found that total T3 (TT3) levels (OR = 0.738; CI: 0.506-1.076; p = 0.1142) were not significantly associated with NAFLD risk. Shi et al. [24] observed that among T2DM patients, FT3 levels were significantly elevated in the NAFLD group (z = -4.07; p < 0.001) compared to the non-NAFLD group. There was an increase in NAFLD prevalence, moving from the lowest FT3 tertile to the highest FT3 tertile (p for trend < 0.001). Moreover, high FT3 levels were associated with higher NAFLD risk (OR = 1.301; 95% CI: 1.028-1.645; p < 0.05) after covariate adjustment for age, gender, BMI, T2DM duration, TSH, FT4, smoking, systolic and diastolic blood pressure as well as indices of islet function, blood glucose, liver function, renal function, and lipid levels.

Thyroid Autoantibodies and Risk of Non-alcoholic Fatty Liver Disease

A total of four studies [17-19,22] evaluated the role of TPOAb in NAFLD risk, of which two studies [17,19] also assessed the role of TGAb. Wang et al. [17] found that T2DM patients with NAFLD had significantly higher TPOAb levels (p < 0.01) as well as higher TPOAb/TGAb ratio (p = 0.02) compared to T2DM patients without NAFLD. TGAb levels were not significantly different (p = 0.19) between the two groups. Moreover, higher levels of TPOAb were significantly (p < 0.01) associated with more severe fatty liver changes on ultrasound. Bano et al. [18] noted that TPOAb levels were not significantly associated with NAFLD risk (OR = 1.09; 95% CI: 0.89-1.32; p > 0.05). Eshraghian et al. [19] observed that neither TPOAb (OR = 0.81; 95% CI: 0.45-1.43) nor TGAb (OR = 0.84; 95% CI: 0.47-1.52) were significantly (p > 0.05) associated with NAFLD. Ludwig et al. [22] observed no significant difference in TPOAb levels (p = 0.4063) in subjects with NAFLD than those without NAFLD.

Discussion

One of the earliest studies to suggest an association between HT and NAFLD was by Liangpunsakul et al. [25] in 2003. Since then, various studies have been conducted to evaluate this relationship of scientific and clinical significance. In this review, we have assessed 10 such relevant studies that met the specified eligibility criteria. The association between HT and NAFLD can be due to overall thyroid dysfunction, the impact of individual thyroid function parameters (TSH, T3, T4, TPOAb, TGAB), or both.

In our review, six studies directly evaluated the effects of hypothyroid status in NAFLD risk. Two of them found that HT (SCH, OH, or both) was an independent risk factor for NAFLD, whereas four of them could not ascertain any such association. Out of the eight studies assessing the independent role of T4, three studies found that reduced T4 levels are associated with NAFLD, while others did not find any such relationship. The difference in findings among these studies can be attributed to the fact that there was a vast heterogeneity in the definition of HT among each of these studies, as shown in Table 1. Although most of these studies diagnosed HT based on high TSH and low T4 levels, the cut-off values for these thyroid function parameters differed among them. Therefore, an individual diagnosed with HT as per one study could be considered euthyroid in another study due to inter-study variation in TSH or T4 cut-off values. Also, patients diagnosed with HT in one [23] of these studies were receiving T4 replacement therapy, which could obscure the effects of functional hypothyroid status in NAFLD pathogenesis. Moreover, the differences in the source of the study population and the sample size itself could have led to different conclusions among each of these studies.

Some explanations have been put forth to explain the lack of independent association between HT and NAFLD. Janovsky et al. [15] have suggested that this relationship may not be independent of metabolic syndrome risk factors. They found that euthyroid patients with elevated TSH levels were 1.22 times more likely to have NAFLD; however, this association became insignificant after adjusting for metabolic syndrome variables. If this were true, HT could just as well be a confounder rather than an independent risk factor for NAFLD. Eshraghian et al. [19] suggested that the thyroid hormone abnormalities (low TSH, low FT3) seen in NAFLD patients could be a result of sick euthyroid syndrome rather than having a causal role in NAFLD pathogenesis itself. Moreover, there is evidence that mild HT might prevent fibrosis progression in rats [26]. However, it is essential to note that many of these studies are either animal studies or cross-sectional, making it difficult to establish or deny causal association.

**Figure 2:**
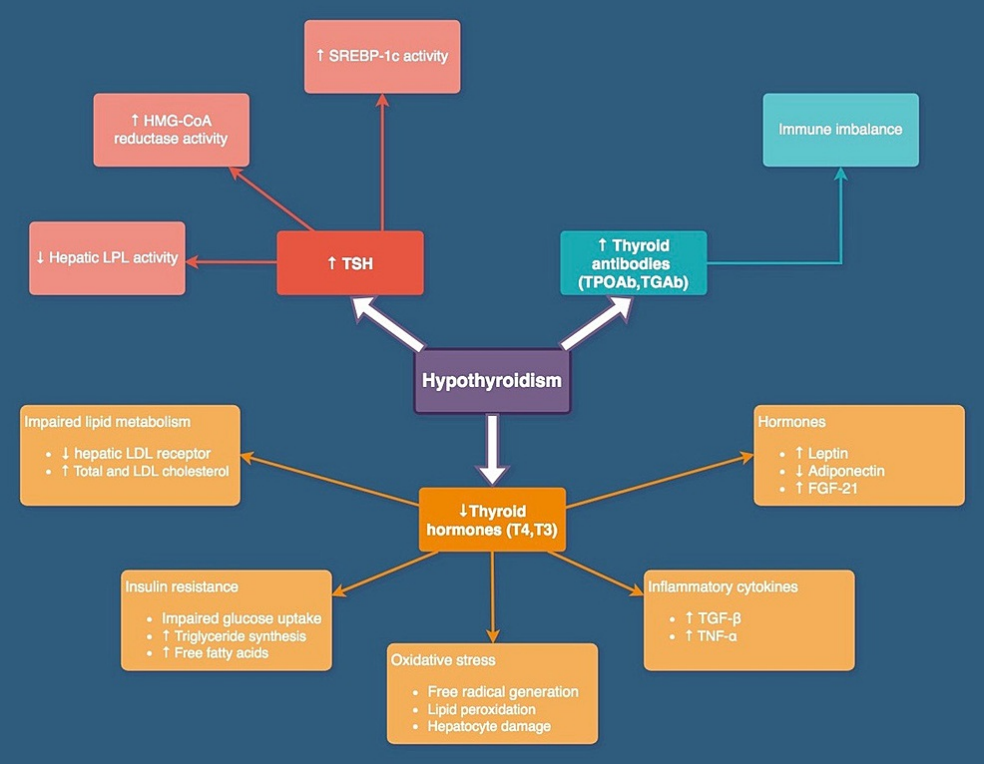
Possible pathophysiological mechanisms of hypothyroidism-induced NAFLD. NAFLD: non-alcoholic fatty liver disease; TSH: thyroid-stimulating hormone; T4: thyroxine; T3: triiodothyronine; TPOAb: thyroid peroxidase antibody; TGAb: thyroglobulin antibody; SREBP-1c: sterol regulatory element-binding transcription factor 1; HMG-CoA: 3-hydroxy-3-methyl-glutaryl-coenzyme A; LPL: lipoprotein lipase; LDL: low-density lipoprotein; TGF-β: transforming growth factor-beta; TNF-α: tumor necrosis factor-alpha; FGF-21: fibroblast growth factor-21

Although not completely understood, NAFLD is thought to result from an interplay of several genetic, environmental, and metabolic factors [27]. As shown in Figure 2, several factors have been implicated in the pathogenesis of HT-induced NAFLD, such as impaired lipid metabolism, insulin resistance, oxidative stress, and the role of inflammatory cytokines and other hormones. Through their effects on hepatic thyroid hormone receptor β, thyroid hormones play an essential role in the sequestration of lipid droplets in hepatocytes, also known as lipophagy, and hence play a crucial role in lipolysis [28]. Lack of TH effect in HT leads to decreased low-density lipoprotein (LDL) receptors on hepatocytes, resulting in impaired cholesterol excretion, elevated apolipoprotein-B levels, and elevated total-cholesterol and LDL-cholesterol levels [29]. Studies have also noted a positive correlation between HT and the high HOMA-IR index, a tool used to represent insulin resistance [23]. Insulin resistance, in turn, leads to impaired glucose uptake, increased triglyceride synthesis, increased peripheral lipolysis, and increased liver free-fatty acid (FFA) uptake by the liver - changes associated with NAFLD [30].

Apart from its effect on lipid and glucose metabolism, HT has also been associated with elevated oxidative stress markers [31]. Mitochondria, classically known as the cell’s powerhouse, play an essential role in β-oxidation of fatty acids, oxidative phosphorylation, electron transfer, and adenine triphosphate generation. Elevated FFA and mitochondrial dysfunction lead to free radical generation [32]. This accumulated reactive oxygen species enhances lipid peroxidation and can lead to hepatocyte damage and activation of pro-inflammatory cytokines. Cytokines such as tumor necrosis factor-alpha and transforming growth factor-beta can then enhance the progression of NAFLD to fibrosis by activating hepatic stellate cells [33,34]. Adipocytokines such as leptin and adiponectin have also been implicated in the multi-hit hypothesis for NAFLD progression to fibrosis [35]. Patients with HT have been observed to have high leptin levels [36]. Leptin, a hormone involved in appetite regulation, increases hepatic insulin resistance by initiating dephosphorylation of insulin receptor substrate-1 [37]. It also promotes collagen synthesis in the liver, hence playing a role in NAFLD progression to fibrosis [38]. Adiponectin, a hormone secreted by adipose tissue, plays a vital role in maintaining hepatic insulin sensitivity [39], and its administration in obese mice has been shown to reduce hepatic steatosis [40]. Apart from these hormones, high levels of another hormone, fibroblast growth factor-21 (FGF-21), have been observed in hypothyroid individuals [41]. Treatment with FGF-21 analog in NASH patients has been found to reduce liver fat content [42], implicating its role in NAFLD pathogenesis.

A total of nine out of ten studies evaluated the independent role of TSH in NAFLD pathogenesis. Of these, five studies could not establish any significant relationship between them, whereas four studies concluded that elevated TSH levels were an important risk factor for NAFLD. Moreover, Bano et al. [18] found that higher serum TSH levels were associated with liver stiffness ≥ 8 kPa on transient elastography, suggesting that TSH levels can be a future marker for liver fibrosis among NAFLD patients. TSH is believed to mediate its impact in NAFLD pathogenesis mainly by affecting hepatic lipogenesis. High TSH levels can lead to elevated triglyceride levels by decreasing hepatic lipoprotein lipase activity [29,43]. The binding of TSH to its receptor on hepatocytes leads to increased expression of genes involved in lipogenesis by initiating hepatic sterol regulatory element-binding transcription factor 1 activity via the cAMP/PKA/PPARa pathway [44]. Moreover, TSH has been found to increase the expression of hepatic 3-hydroxy-3-methyl-glutaryl-coenzyme A reductase (HMG-CoA reductase), a rate-limiting step in cholesterol biosynthesis [45]. Moreover, TSH also accelerates lipolysis and increases FFA levels [46], which are known to play a role in free radical generation [32].

Of the four studies analyzing T3 levels, one study found that FT3 levels are significantly higher in NAFLD patients than non-NAFLD patients, whereas others observed no such relationship. The difference in the observed results could be due to the difference in the study population chosen, with hospitalized T2DM patients chosen in Shi et al. [24] versus the relatively healthy sample population in the other three studies [19,20,22]. Shi et al. [24] proposed that the high FT3 levels observed in NAFLD patients in its study could be due to disruption of the hypothalamic-pituitary-thyroid axis. On the other hand, Ludwig et al. [22] proposed that the lack of association between TT3 levels and NAFLD observed in its study could be due to inhibited conversion of TT4 to TT3. Furthermore, Gokmen et al. [20] concluded that a high FT3/FT4 ratio, an indicator of peripheral deiodinase activity, was significantly associated with NAFLD risk. It has been suggested that this could be a compensatory mechanism to increase energy expenditure in obese individuals [47]. Out of the four studies evaluating the role of thyroid autoimmunity, one study found that high TPOAb titers and high TPOAb/TGAb ratio were associated with high NAFLD risk. As some autoantibodies such as antinuclear antibodies have been noted in NAFLD patients, thyroid autoimmunity and immune imbalance, suggested by high TPOAb levels, could play an etiological role in NAFLD pathogenesis [48]. Further studies could aid in elaborating the specifics of this role of TPOAb and TGAb, if any. However, on the other hand, it is essential to note that Wang et al. [17] chose hospitalized T2DM patients as subjects; and this role of TPOAb may not be relevant in non-diabetic subjects.

Because NAFLD accounts for one of the most common causes of chronic liver failure worldwide and its incidence is increasing steeply, it is essential to make every potential attempt to understand its various possible etiologies better. Here, we have presented an updated review to represent the current evidence on HT-induced NAFLD and explained the possible pathophysiological mechanisms. As the current therapeutic drugs for NAFLD treatment primarily involve non-specific agents and some clinical trials have observed the beneficial role of thyroid hormone analogs [49] in reducing NAFLD-related steatosis, understanding this role of HT in NAFLD could lead to a clinical breakthrough in NAFLD management. However, because contradictory results are found in different studies, and no accord has been reached so far, several questions remain unanswered. Is the relationship between HT and NAFLD causal or only a co-existing finding? Is this supposed association independent of metabolic syndrome risk factors or a mere confounder? If independent, does HT only play a role in causing NAFLD or also in its progression to NASH, cirrhosis, and fibrosis? We recommend that long-term prospective studies with a large sample size be further conducted to prove a causal relationship, if any. In addition, clinical trials can be conducted to elucidate if thyroid hormone replacement can halt or reverse NAFLD progression.

Although we have attempted to take an unbiased, objective stand in our systematic review, it is not free from limitations. As there is wide heterogeneity in the study population characteristics, outcomes assessed, and covariates adjusted in each of the included 10 studies, a direct comparison is complex, and hence it is not easy to draw any conclusion. Also, nine out of ten studies diagnosed NAFLD based on ultrasonography, which can miss mild steatosis, leading to underestimation of study findings. Moreover, as we have only included articles with free full text available, we could have missed some critical studies in our review. Finally, seven out of ten included studies were cross-sectional and therefore lacked the temporality required to establish a causal association.

## Conclusions

As the worldwide clinical and financial burden of NAFLD is rapidly rising, we have attempted to analyze the evidence so far in the etiological role of HT in NAFLD pathogenesis. In our systematic review, some studies noted that HT or thyroid function parameters (TSH, T4, T3, TPOAb) were significantly associated with NAFLD, while others could not ascertain any such relationship. There was a wide heterogeneity in the study population characteristics, sample size, diagnostic approach, and thyroid function parameters assessed among each of the included studies, making a direct comparison difficult. If this association between HT and NAFLD is proven to be causal, it could open a new window of opportunity, not only for NAFLD treatment but also for its screening and prevention. We recommend that extensive, long-term prospective studies assessing key thyroid function parameters be conducted to identify any causal association between HT and NAFLD. Moreover, placebo-controlled, randomized clinical trials could be carried out to determine if thyroid hormones or their analogs can effectively mitigate the fatty liver changes in NAFLD and prevent its progression.
